# Subknots in ideal knots, random knots, and knotted proteins

**DOI:** 10.1038/srep08928

**Published:** 2015-03-10

**Authors:** Eric J. Rawdon, Kenneth C. Millett, Andrzej Stasiak

**Affiliations:** 1 University of St. Thomas, Department of Mathematics, Saint Paul, MN, USA; 2 University of California Santa Barbara, Department of Mathematics, Santa Barbara, CA, USA; 3 University of Lausanne, Center for Integrative Genomics, Faculty of Biology and Medicine, Lausanne, Switzerland; 4 Swiss Institute of Bioinformatics, CH-1015, Lausanne, Switzerland

## Abstract

We introduce disk matrices which encode the knotting of all subchains in circular knot configurations. The disk matrices allow us to dissect circular knots into their subknots, i.e. knot types formed by subchains of the global knot. The identification of subknots is based on the study of linear chains in which a knot type is associated to the chain by means of a spatially robust closure protocol. We characterize the sets of observed subknot types in global knots taking energy-minimized shapes such as KnotPlot configurations and ideal geometric configurations. We compare the sets of observed subknots to knot types obtained by changing crossings in the classical prime knot diagrams. Building upon this analysis, we study the sets of subknots in random configurations of corresponding knot types. In many of the knot types we analyzed, the sets of subknots from the ideal geometric configurations are found in each of the hundreds of random configurations of the same global knot type. We also compare the sets of subknots observed in open protein knots with the subknots observed in the ideal configurations of the corresponding knot type. This comparison enables us to explain the specific dispositions of subknots in the analyzed protein knots.

Studies of 3-D trajectories of polypeptide chains forming knotted proteins reveal that more complex knots frequently contain simpler knots and slipknots^1^,^2^,^3^. For example, some subchains of a static configuration polypeptide chain forming the 6_1_ knot can be classified as forming the 4_1_ knot, while a polypeptide chain forming the 5_2_ knot has subchains forming 3_1_ knots^3^. It seems reasonable that as a portion of a knotted chain is shortened, the associated knot type should be progressively simplified until reaching the unknot, 0_1_. However, why subchains of 6_1_ knots should form 4_1_ knots and subchains of 5_2_ knots should form 3_1_ knots is much less evident. Here we study the question: “What are the knot types of the subchains that are contained in a configuration of a complex knot type?” We call the knot types arising from subchains *subknots* of the configuration. Although this question was stimulated by studies of linear knots formed by the polypeptide chains of knotted proteins, we study it here for subknots formed in two special classes of closed chains: the KnotPlot chains [Scharein, R. G. *KnotPlot*. (1998) Available at: http://www.knotplot.com/] which visually reflect the structural regularity of the classical prime knot presentations and preserve the knot types' symmetries^4^,^5^ and the ideal knot configurations^6^,^7^,^8^,^9^,^10^,^11^,^12^,^13^ whose structural properties reflect the spatial nature of knotted magnetic flux lines and of knotted macromolecules^6^,^14^,^15^,^16^,^17^,^18^,^19^,^20^,^21^. We compare the sets of subknots to the knot types obtained by changing crossings in minimal knot diagrams for the knot types, the so-called predecessor knots^22^,^23^. We then compare the subknots seen in these regular configurations to the subknots seen in random configurations. Building upon this study, we consider linear polypeptide chains and discuss what the resulting information tells us about the presence of certain knotted subchains within a knotted polypeptide chain.

## Results and Discussion

### The disk matrix reporting the knot type of every subchain in a closed chain

**Figure 1:**
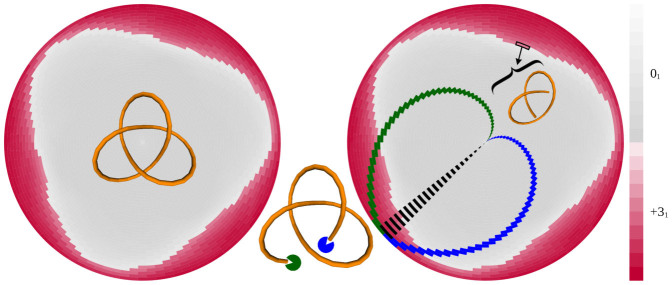
A guide to disk matrices reporting the knot type of every subchain in a polygonal chain forming a KnotPlot trefoil knot. The left panel shows the disk matrix obtained for the polygonal axial trajectory of a symmetric configuration of a trefoil knot (shown in the center of the matrix). Similar matrices for other knots are presented in Figs. 2–6. The right panel and the drawing between the two panels are intended to explain the principle of the matrix. For the explanation see the main text. The underlying brick wall pattern of the matrix is a consequence of the fact that the longitudinal position of the cell indicates the position of the center vertex of the represented chains. For subchains with even numbers of segments, the centers of these subchains fall at a vertex. For subchains with odd numbers of segments, the centers lie at the middle point of a segment.

Taylor and later King et al. introduced square and triangle-shaped matrices in which the cells report the knot types of the subchains of a linear chain^2^,^24^. This type of matrix, however, does not adequately reflect the circular periodicity found in a closed chain and, thus, is not well-suited to report the knot types of subchains for closed, circular chains. To overcome this problem, we introduce a disk matrix (see Fig. 1) that reports the knot type of every subchain in a fixed embedding of a circular polygonal chain. It is helpful to think of the disk matrix as being composed of cells delimited by longitude lines radiating from the center of the disk and concentric latitude circles with increasing radius. The matrix cells close to the center of the disk represent very short subchains (starting from one segment), whereas cells bordering the rim of the disk represent long subchains (missing just one segment). The longitudinal position of a cell indicates the position of the midpoint of the associated subchain and the latitude indicates the length, in number of segments, of the associated subchain. A chain composed of 100 segments, for example, has a matrix with 99 latitude and 100 longitude lines where the Greenwich longitude (i.e. positive *x*-axis) corresponds to subchains whose center point is the first vertex of the polygonal chain. The numbering of longitude lines goes in a counter-clockwise direction in our matrix. Colors of the cells in the matrix indicate the dominant knot type of the corresponding subchains, i.e. the knot type most frequently resulting from a uniform closure procedure of the open chain (see Refs. 3, 25,26,27,28 and the Materials and Methods section). The intensity of a given color reflects how frequently this dominant knot type occurs among the tested closures^3^,^28^. Fig. 1 shows the disk matrix reporting the two knot types occurring in subchains of the symmetric trefoil knot configuration shown in the center of this disk matrix. The polygonal trefoil knot, 3_1_, consists of the center-lines of 47 cylindrical segments. This trefoil configuration has a three-fold rotational symmetry that one also can see in the symmetry of the disk matrix. Near the center of the matrix, we have entries corresponding to short subchains. These entries are colored gray (see the color scale at the right of Fig. 1) indicating that the dominant knot type is the unknot, 0_1_, for the closures of these short subchains. As one moves away from the center and closer to the edge of the disk matrix, the individual cells represent subchains that have sufficient length to form trefoil knots as the dominant knot type upon closure. These cells are colored red to indicate the trefoil knot. Notice that cells close to the border between the zones of the 3_1_ knot and the unknot have colors of decreased intensity (red and gray, respectively). This border effect indicates that the corresponding subchains show a decreasing preference to form the indicated knot types as the closures also create increasing numbers of other knot types, for example those knot types that dominate the other side of the apparent border. Since the knot configuration, a KnotPlot trefoil, is spread out it takes quite a bit of length to realize the global knot type. Later we analyze random configurations, in which the subknots are more localized (i.e. the coloring starts much closer to the center of the disk matrices) and where there is a more diverse spectrum of subknots. Note that we only see unknot and trefoil subchains in this highly regular trefoil knot due to its relatively simple spatial structure.

For each knotted configuration, there is a shortest length at which the global knot is realized. The minimal length subchain or subchains realizing the host knot is called the knot core^29^ and is usually determined by the cell(s) closest to the center of the shortest subchain having the global knot type. In the right panel of Fig. 1, one such cell corresponding to a knot core is outlined in black with the corresponding subchain shown nearby. In this panel, the cells colored blue and green represent the subchains resulting from progressively shortening the subchain from each end one segment at a time (represented by the blue and green “pacmen” in the central figure) starting from the same initial scission. The black cells represent the result of simultaneously removing a segment from both ends, thereby shortening the chain by two at each step and giving the dashed pattern. Note that the centers of the chains resulting from progressively removing segments from one end define a spiral path moving from the rim global knot to the center unknot. The direction of the spiral reflects the choice of the end that is being trimmed.

### KnotPlot configuration subknots and predecessor knots

**Figure 2:**
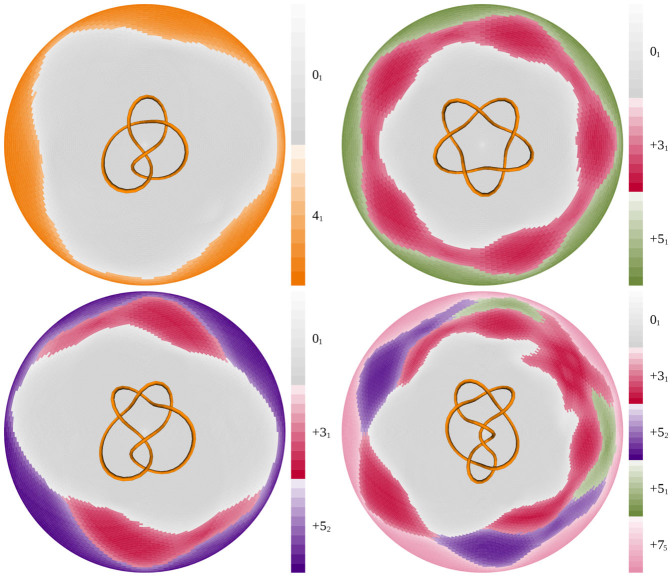
Disk matrices for KnotPlot configurations of the 4_1_ (A), 5_1_ (B), 5_2_ (C) and 7_5_ (D) knots. The KnotPlot configurations of the corresponding knots are presented over centers of their disk matrices.

Fig. 2 shows disk matrices for closed chains forming several other knot types. These knotted chains are configurations created using KnotPlot and are configurations resulting from computations that mimic the action of Coulomb forces on charged elastic fibers forming a given knot type. We chose this group of knot configurations for our initial study because they reflect symmetries of the knot types and the configurations have a projection that looks very similar to the minimal crossing diagrams of the knot types^4^,^5^. We analyze the polygonal configurations determined by the centerlines of these tubes, taking into account that our tubes are not smooth but composed of many small cylinders.

We continue our analysis with the KnotPlot figure-eight knot, 4_1_ (Fig. 2A). The figure-eight knot, 4_1_, is a twist knot having unknotting number one^5^, as does 3_1_ (which is both a twist knot and a torus knot). Thus one crossing change can change directly either of them to the unknot^30^,^31^,^32^. This feature is visible in the disk matrix by the direct passage from the global knot type to the gray colored zone as the length of the subchains gets shorter.

Fig. 2B shows the disk matrix for the 5_1_ knot, another torus knot. Subchains of the 5_1_ knot are capable of forming the 3_1_ knot type. Of course, subknots forming the unknot are always observed since any polygon with fewer than six edges (and, thus, subchain with four or fewer edges) is unknotted^33^. The KnotPlot configuration of the 5_1_ knot has a toroidal five-fold symmetry that can be seen in its disk matrix. The unknotting number of the 5_1_ knot is two, which also is visible in the disk matrix, since to pass from the green colored 5_1_ zone to the zone where the subchains only form unknots, one needs to pass through the zone of subchains forming 3_1_ subknots.

The next example (Fig. 2C) is the 5_2_ knot, a twist knot (as are 3_1_ and 4_1_). Twist knots always have unknotting number equal to one. Again, as in the case of disk matrices for the 3_1_ and the 4_1_ knots, one can pass directly from the global knot zone to the unknot zone. One also can pass through the 3_1_ intermediate zone on the way to the zone of unknots. The disk matrices we have computed for the KnotPlot configurations (and later ideal knots) with the unknotting number equal to one always showed a direct passage from the zone of the global knot to the zone of the unknot. It is tempting, therefore, to conjecture that this is always the case for knot types with the unknotting number one. The disk matrix of the 5_2_ knot shows that, as the chain forming the 5_2_ knot is shortened, it can transition either to a 3_1_ knot or to an unknot. This resembles the situation in which a minimal diagram of the 5_2_ knot is subject to single crossing changes^22^. The knots resulting from single crossing changes are either 3_1_ knots or unknots.

Knot types arising from individual crossing changes performed on a minimal crossing diagram of knot type 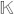 have been called *predecessors* of 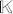
22 since they typically have a smaller minimal crossing number than the knot type 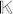. To be more precise, in Ref. 22 the objective was to distinguish predecessors of various generations arising from the classical knot presentations. The first-generation predecessors are the knot types that are obtained by a single crossing change in a minimal crossing diagram of a given knot, whereas the second-generation predecessors are obtained by single crossing changes performed on minimal diagrams of the first-generation predecessors, etc. Diao et al.^23^ showed that starting from any minimal diagram of a given alternating knot, one always obtains the same set of first-generation predecessors due to that fact that any such diagram is related to any other by a simple transformation known as a “flype”. For non-alternating knot types, different minimal diagrams can produce different sets of first-generation predecessors. As a consequence, the sets of predecessors for non-alternating knot types depend on the actual knot diagrams chosen and therefore the set of predecessors for non-alternating knot types is not a topological invariant. For this reason, we focus our analysis on alternating knot types that do not have non-alternating predecessors.

The knot 7_5_ was specifically discussed by Diao et al.^23^ and was shown to have 3_1_, 5_1_, and 5_2_ knots as first-generation predecessors. The second-generation predecessors arising from a single crossing change in minimal diagrams of 5_1_ knots are 3_1_ knots, those arising from 5_2_ knots are 3_1_ knots and unknots, and those arising from 3_1_ knots are always unknots. Finally, the third-generation predecessors arising from the 3_1_ knots that have come from 5_1_ or 5_2_ subknots are also unknots. Fig. 2D shows the disk matrix of the KnotPlot configuration of the 7_5_ knot. We see that the 3_1_, 5_1_, and 5_2_ knots also form first-generation subknots. First-generation subknots can be recognized easily in the disk matrices as having territories that can be accessed directly from the territory of the global knot while advancing radially toward the center of the matrix. We also see that the 3_1_ knot, in addition to being a first-generation subknot, is a second-generation subknot that arises by truncating subchains forming the 5_1_ and 5_2_ knots. Finally, we see that unknots can emerge as second- or third-generation subknots from first- or second-generation subknots, respectively. Interestingly, the disc matrix of the 7_5_ knot also indicates the predecessor knots which are more likely to appear after randomly changing a crossing in a minimal crossing diagram of the 7_5_ knot. The 5_2_ subknots share the longest border with the global knot 7_5_ and three of the seven crossing changes to the 7_5_ minimal diagram result in 5_2_ knots. Meanwhile, the 3_1_ and 5_1_ predecessors each appear in two of the seven crossing changes^23^.

**Figure 3:**
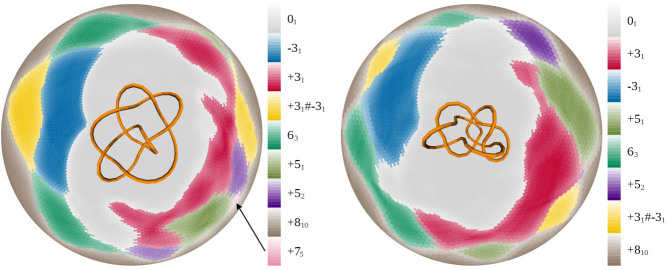
Comparison of disk matrices for KnotPlot (A) and ideal knot (B) configurations of the 8_10_ knot. Notice that the 7_5_ knot is visible as one of the subknots in the disk matrix of the KnotPlot configuration (indicated with an arrow), whereas the ideal knot configuration does not contain this subknot. The KnotPlot and ideal knot configurations of the 8_10_ knot are shown over the center of their disk matrices.

Encouraged by the observed degree of agreement between the subknots and the predecessor knots coming from the minimal crossing diagrams, we compared the KnotPlot configuration subknots to the set of predecessors of all knot types with up to 10 crossings for which the set of predecessors is defined (see above). For knot types with up to seven crossings, the set of observed subknots (of all generations) correspond to the set of predecessor knots (of the corresponding generation). However, as the knots increase in complexity, there is an increasing number of cases where one observes subknots that are not present among the set of predecessors as well cases where some of predecessor knots are not present among the subknots (see Table 1). Interestingly, the predecessor knots that are not present among the subknots belong to the predecessors of second and higher generations. We will discuss later how we might find these higher order predecessors within these configurations. The 8_10_ knot (Fig. 3A) is the first example where we see subknots that are not predecessors. In addition to the predecessors 6_3_, 3_1_#–3_1_, 5_1_, 5_2_ 3_1_, −3_1_ and 0_1_, the KnotPlot 8_10_ configuration also contains a 7_5_ subknot (indicated with an arrow).

### Ideal configuration subknots

Ideal knot configurations are defined by the axial trajectories of uniform diameter tubes that reach the minimum length necessary to form a given knot type^6^,^7^,^8^,^9^,^10^,^11^,^12^,^13^ and have been shown to have properties that correspond to those found in knotted magnetic flux lines and knotted macromolecules^6^,^14^,^15^,^16^,^17^,^18^,^19^,^20^,^21^. Visually, one observes that these configurations are more compact than KnotPlot configurations as a consequence of the minimization of the amount of “rope” used to create the knot.

Fig. 3B shows the disk matrix for the ideal 8_10_ knot. All of the predecessor knot types (i.e. 6_3_, 3_1_#-3_1_, 5_2_, 5_1_ +3_1_, −3_1_ and 0_1_) occur while the 7_5_ knot does not occur.

This result suggests that the reduction of the 3-D trajectory to the necessary minimum required to build a given knot reduces the presence of subknots which are not predecessors. Indeed we observe fewer non-predecessor subknots in ideal configurations than in KnotPlot configurations. However, three 10-crossing knot types (10_69_, 10_97_, and 10_114_) have subknots that are not predecessors. For example, the ideal (10_69_) has a subknot 7_3_ that is not among the predecessors of that knot. Analyzing this case more closely we noticed that although there are subchains of the ideal 10_69_ configuration that form 7_3_ knots more frequently than any other knot types upon the uniform closure procedure, the fraction of closures forming 7_3_ knots is around 20%. This observation prompted us to consider a more discriminating class of subknots, which we call the *majority subknots*, consisting of knot types that are formed in at least 50% of the closures for some subchain. Interestingly, all of the majority subknots observed in the analyzed ideal and KnotPlot configurations belong to the set of predecessors of the corresponding knot types. We then analyzed whether all predecessor knots are observed among the majority subknots of ideal knots. Some predecessors are not represented amongst the majority subknots but only for predecessors of second and higher generations. All first-generation predecessors are present among the majority subknots of ideal knots. This is not the case, however, for the KnotPlot configurations where some of the first-generation predecessors do not reach the strict criterion of 50% closures (see Table 1).

Among KnotPlot and ideal knot configurations of all prime knots through 10 crossings, only 3_1_ and 4_1_ do not contain subknots other than the global knot and the unknot. Furthermore, all KnotPlot and ideal knot configurations contain either a 3_1_ or 4_1_ subknot.

### An analysis of second- and higher-generation predecessors and subknots

**Figure 4:**
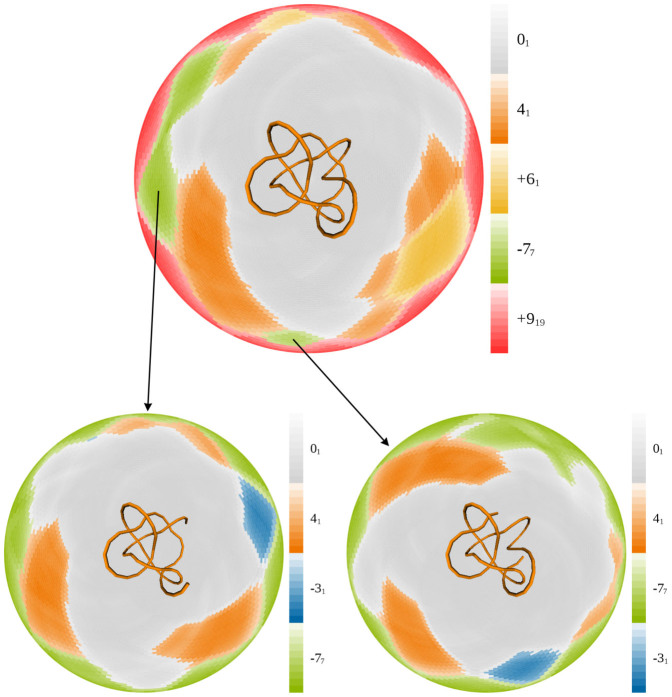
A procedure that reveals all second-generation predecessors. Two different subchains that form −7_7_ knotted arcs in the ideal configuration of the 9_19_ knot are closed using the underlying idea of closure at infinity whereby two parallel rays are placed at the endpoints of the analyzed subchain. Instead of extending the rays to infinity, the rays are cut as soon they leave the the convex hull of the analyzed subchain and then are closed with an additional segment, yielding a configuration equivalent to the closure at infinity. The corresponding subchains are shown at the center of the corresponding disk matrices. After checking that the closure produced the desired −7_7_ knot, the polygonal chains were analyzed to determine their subknots. Note that, in each case, the “extracted” −7_7_ knot contains −3_1_ subknots even though the −3_1_ is not among the subknots of the ideal 9_19_.

In all but one of the ideal configurations of knot types with nine or fewer crossings for which the predecessors are defined, 67 in total, we found that the set of predecessor knots and the set of subknots of the ideal configurations were the same. Fig. 4 shows the disk matrix of the one exceptional case, an ideal 9_19_ knot. This positive 9_19_ knot has a −7_7_ knot as one of its first-generation subknots. The predecessors of the −7_7_ knot are 4_1_, −3_1_, and 0_1_. However, the −3_1_ subknot is not observed as a second-generation subknot of the ideal 9_19_ configuration.

We are, therefore, led to ask, “Why do the first-generation subknots usually agree with the subknots of ideal configurations but not always those of the second-generation?” This behavior, at least to some extent, comes from differences in the approaches of determining predecessors versus subknots. In particular, predecessors are obtained by a distributive process whereby one crossing is changed in the minimal diagram and then the minimal diagram for the new knot type is analyzed to find the second-generation predecessors. This process is akin to changing one crossing and then changing any other crossing. On the other hand, the analysis of subknots only looks at subchains that are obtained by further trimming subchains that form the given subknot. Thus, the subknot search can be thought of as being a processive process since removing subarcs of increasing length behaves like removing nearby crossings in an ordered fashion as one moves through the configuration. The distributive and processive processes differ in important ways. For example, one does not investigate the subknots that could be revealed if the chain were trimmed at two different portions of the knot. Of course, we cannot open the chain at two (or more) different places using the uniform closure technique^3^,^28^ because there would be four (or more) endpoints of the chain.

To simulate the distributive process, we analyzed the ideal configuration of the 9_19_ knot to see if we could find the second-generation −3_1_ knot that emerges from the first-generation −7_7_ predecessor. We took one representative −7_7_ knotted subarc from each of the two regions of the 9_19_ that were shown to be −7_7_ knots. The regions and the configurations are seen in Fig. 4. We then closed each of the two configurations in one of the closure directions that yields a −7_7_ knot and did our subarc analysis on these configurations. We see that both configurations indeed contain −3_1_ subknots. We used this procedure to search for eight different second- and higher-generation predecessors that did not appear as subknots in the disk matrices for ideal configurations. In each case the distributive process, such as the one shown in Fig. 4, revealed the predecessors as subknots of lower order subknots.

### Analysis of the subknots found in random configurations of a given knot type

With the examples above, we have developed an understanding of subknots arising from the classical knot projections, from KnotPlot knots, and from ideal knots. We now ask: “In random configurations, is there a common set of subknots for a given knot type? Furthermore, is the set of subknots related to the set of subknots and predecessors from our previous analysis?” Of course, in the case of random configurations, we expect many different subknots, but could there be a common set?

We generated 100,000 random equilateral polygons composed each of 100 segments and analyzed the configurations forming eight or nine crossing knot types that we had analyzed. We chose eight and nine crossing knot types because they have a number of subknots/predecessors and sufficiently large sample sizes.

**Figure 5:**
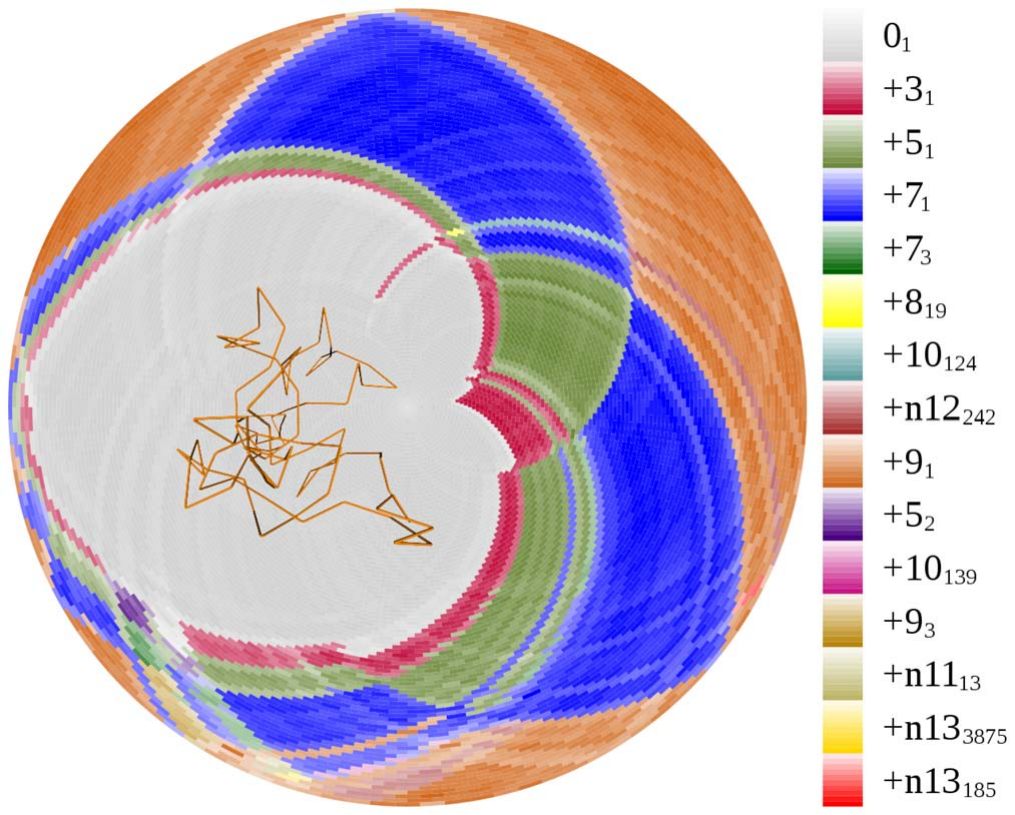
A random 9_1_ knot and its disk matrix. Notice the presence of the predecessors 7_1_, 5_1_ and 3_1_ as first, second, and third-generation subknots, respectively. Notice also the presence of several more complicated subknots, which consume a small amount of the area of the disk matrix. For the knot types with more than 10 crossings, we use the Dowker-Thistlethwaite notation where the leading letter (*n* or *a*) tells whether the knot type is non-alternating or alternating, respectively, and the knot types are indexed within the non-alternating and alternating classes.

We start the discussion of random conformations with an analysis of random configurations forming the 9_1_ knot type. The ideal subknots, KnotPlot subknots, and predecessors are all 7_1_, 5_1_, 3_1_, and 0_1_ and we detected 27 configurations of 9_1_ knots (right or left-handed). Each of these configurations showed the presence of all of these knot types as subknots although, as expected, a number of additional subknots are also visible. Fig. 5 shows one of these random 9_1_ knots and its associated disk matrix. We see that the 7_1_ knot occurs as a first-generation subknot from which 5_1_ subknots emerge and which, in turn, give rise to 3_1_ subknots. We also see additional knot types, some of which have a higher minimal crossing number than the global knot. These more complicated subknots frequently arise as subknots in random configurations but appear for only very shorts intervals of length and are visible only on a small total area of the disk matrix. Furthermore, the more complicated subknots do not appear as subknots of the KnotPlot or ideal configurations and thus are specific to the random configurations instead of being potentially conserved. In the great majority of the configurations of the random non-trivial knots, we see all of the ideal subknots. For example, in each of the 228 configurations of 8_1_ knots, we always saw the subknots 6_1_, 4_1_, and 0_1_. And, in each of the 220 random configurations of 8_2_ knots, we always saw the subknots 6_2_, 5_1_, 4_1_, 3_1_, and 0_1_.

There are a total of 3334 samples from eight-crossing knot types and 1451 samples from nine-crossing knot types, for a total of 4785 samples. Of these, 4697 (≈98.16%) of the samples contained the ideal subknots of all generations. When some of the ideal subknots are not detected in the random configurations, we believe that this often is caused by the fact that the random configurations have many edges that pass very close to other edges. Our analysis will have difficulty detecting all of the transitions in such situations because we only analyze subarcs ending at vertices. Therefore, the removal of a single segment may have the effect of changing several crossings and we can pass directly to higher generation subknots.

While the KnotPlot subknots (90.91%) and predecessors (98.04%) both have good agreement with the subknots of random configurations, the best agreement is with the ideal subknots (98.16%). For the random configurations of some knots types, there are only a few cases where an ideal subknot is not present in a random configuration and we can examine whether refining the image will show the additional knot types. For example, among 228 configurations of ±8_4_ knots, we found only one configuration that did not show each of the ideal subknots after our standard analysis. When we repeated the analysis after dividing each segment of the same configuration into five equal size segments, we detected the subknot.

There are, however, instances where this finer resolution did not reveal all of the ideal subknots. The most striking case of this phenomena was provided by random configurations of 8_16_ knots, where 15 out of the 64 analyzed configurations did not contain the first-generation 5_2_ knot. Refinement helped in only one case but not in the remainder of the cases. Therefore, this suggests that at least for some alternating knots, it is possible to have configurations that do not show all of the ideal subknots, and in particular even the ideal first-generation subknots. We note that all 64 analyzed configurations of the 8_16_ knots showed the presence of the remaining first-generation predecessors, i.e. 6_3_, 4_1_ and 3_1_. Thus it is possible that the majority of the predecessors are always present in any random configuration of a given knot type but others may occur less frequently. On the other hand, even if thousands of tested random configurations of some knot type show a common set of subknots, it is possible that there are rare configurations of this knot type that do not contain all of these subknots. While the predecessors and subknots of ideal configurations appear to be good predictors of subknots in random configurations, it is an open question as to whether there even is a common set of subknots for all configurations of a given knot type. If there is a common set of subknots and it does not match the predecessors or ideal configuration subknots, is there some other way to describe them?

### What can circular chain subknots tell us about protein knots?

**Figure 6:**
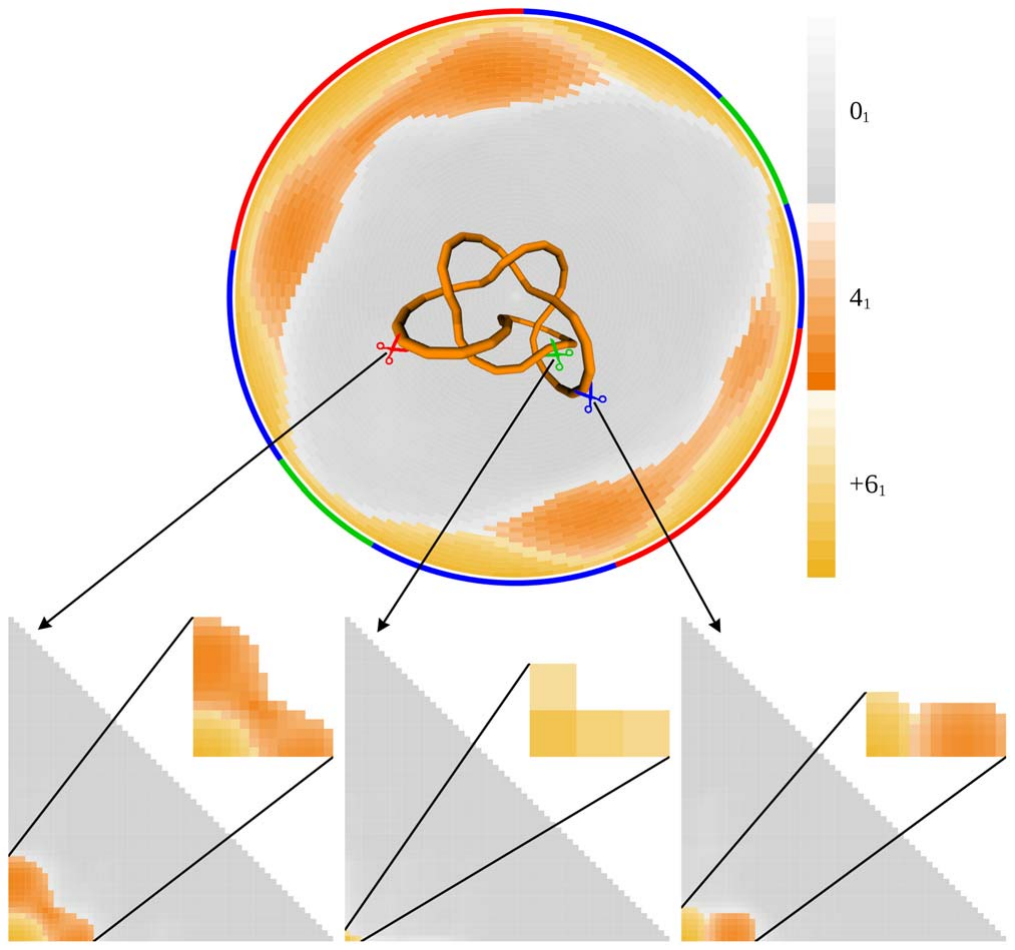
The set of observed subknots in triangular matrices, such as those used to analyze the location of knots in proteins, depends on the position of the segment removed from the analyzed knot. The disk matrix of the ideal configuration of the 6_1_ knot and three triangular matrices calculated for the linear fragments resulting from cleaving at the indicated spots of this 6_1_ knot are shown in this figure. The red, green, and blue arcs near the edge of the disk matrix correspond to the regions within the knot from which the initial scissions lead to the formation of linear fragments giving the qualitatively different triangular matrices shown here. Scissions within red arcs give transitions from 6_1_ to 4_1_ upon trimming of either end. Scissions within green arcs give direct transitions from 6_1_ to 0_1_ upon trimming of either end. Scissions within blue arcs give transitions to the 4_1_ knot upon trimming of one end and a transition to 0_1_ upon trimming the other end. The colors of the scissors indicate the color of the arc within which the scission is located.

Triangle and rectangle-shaped matrices have been used to report the knot type of the subchains of linear chains, e.g. in the analysis of polypeptide chains in searching for knotted and slipknotted proteins^2^,^3^,^24^. In the case of more complex proteins structures forming 6_1_ and 5_2_ knots, trimming the entire polypeptide from its natural C-terminal-end produces non-trivial subknots 4_1_ and 3_1_, respectively, whereas trimming from the N-terminal end immediately produces unknotted subchains^3^. Does such a difference between directions of trimming also occur in the case of circular knots? If so, what is the position of an opening that would allow trimming in one direction to produce different subknots than trimming in the other direction? Fig. 6A shows the disk matrix for the ideal configuration of the 6_1_ knot.

Analyzing the disk matrix of the 6_1_ knot, we see that initial scissions can have three different consequences when the knot is trimmed in the two different directions. There are regions where trimming from either end of an initial scission results in passages from the 6_1_ global knot to the 4_1_ subknot before passing to the unknot. These regions are indicated with a red arc near the rim of the matrix in Fig. 6. There are other regions (green) where trimming from either end results in a direct passage from the 6_1_ global knot to unknots. Finally, there are regions (blue) where trimming of one end results in a passage to the 4_1_ subknot whereas trimming of the other end results in a passage to the unknot. We also indicate the location of the representative cuts for these three categories of regions in the configuration of this 6_1_ knot. For a scission in each of these regions, we computed the triangular matrix used in the analysis of knottedness of linear chains such as those formed by the knotted proteins^3^. One can see that one of the three categories of scissions leads to the situation in which trimming of one end creates a 4_1_ subknot before an unknot is created, whereas trimming of the other end results in a direct passage from a 6_1_ knot to the unknot (see the right triangular knotting matrix in Fig. 6). Note that the regions where the initial scission followed by trimming of one end results in a 4_1_ subknot whereas trimming of the other end results in unknots are placed where the interwound regions end and the apical loops begin. For the ideal 5_2_ knot, there are also three different regions where initial scissions can result in qualitatively different triangular matrices.

A comparison of the triangular matrices obtained after processive trimming of proteins forming 6_1_ and 5_2_ knots (the 6_1_ case is shown in Fig. 6) provides deep insights into the protein structure. With respect to subknots observed by trimming either end, proteins forming 6_1_ and 5_2_ knots produce triangular matrices resembling those observed in ideal knots in which one of the terminal loops is cut so that one end still passes through the other terminal loop. Similar conclusions regarding the location of the polypeptide ends in these proteins have been reached by direct analysis of the knotted protein structures^3^. However, it has not yet been recognized that this particular location of ends is required to achieve the critical property that the trimming of one protein end leads to direct passage to trivial subknots, whereas trimming of other end leads to passages to 4_1_ or 3_1_ subknots, respectively, as is the case of proteins forming the 6_1_ and 5_2_ knots^3^.

## Methods

The disk matrices for a given polygon are created as follows. For each open arc of the polygon (i.e. consecutive set of edges), we create a number (either 100 or 20) of closed polygons and determine the knot type for each of these closures. The numbers 20 and 100 correspond to the number of closure directions analyzed. To make one closure for an open arc, we create a long line segment (designed to move well outside of the convex hull of the original knot) and place the line segment at both of the arc's endpoints. We then connect the free ends of these added segments to create a closed knot. The 20 directions correspond to the vertices of a dodecahedron. In such a case, the knot type corresponding to each direction is weighted by 0.05 since the dodecahedron is perfectly uniformly distributed. For the 100 directions, we generated a roughly uniform set of points/directions on the unit sphere using Martin's polyhedra^34^. We then computed the Vornoi diagram of that point set on the sphere and weighted each direction based on the percentage of the unit sphere consumed by the Vornoi cell containing the direction. For the ideal, KnotPlot, and random configurations, we used 100 closures. When we refined some of the random polygons for closer analysis, we used 20 closures and then verified the results in the critical regions using 100 closures.

The knot types of the closures are determined using a combination of two techniques. We first use software written by one of the authors (EJR) to encode the crossings of a projection of the polygon. Next the crossing code is simplified (potentially) using Thistlethwaite's unraveller program [Thistlethwaite, M. *unraveller*. (2004) Personal communication]. We then compute the HOMFLYPT polynomial^35^ using Ewing and Millett's lmpoly program^36^. Note that the HOMFLYPT polynomial is not a perfect knot invariant (i.e. multiple knot types can share a common HOMFLYPT polynomial^35^). We look up the chiral knot types corresponding to the given HOMFLYPT polynomial via a pre-computed table of polynomials. Secondly, we use Thistlethwaite's knotfind algorithm [Hoste, J. & Thistlethwaite, M. *Knotscape*. (1999) Available at: http://www.math.utk.edu/~morwen/knotscape.html], which generates a “canonical Dowker code”, that we can look up in other tables, to determine a knot type. The knotfind computation is not sensitive to chirality but determines the exact non-chiral knot type. Combining the HOMFLYPT and knotfind information, we can determine the exact chiral knot type for most configurations of knot types with 16 or fewer crossings.

In this study, we analyzed ideal knots, KnotPlot knots, and random knots. The ideal knot configurations were computed using the software Ridgerunner [Cantarella, J., Piatek, M. & Rawdon, E. *Ridgerunner*. (2012) Available at: http://www.jasoncantarella.com/wordpress/software/ridgerunner/], a constrained gradient descent algorithm^12^ for minimizing the ropelength^9^,^11^,^37^,^38^ of a knotted polygon. To reduce the computation time, the ideal configurations from Ref. 12 were reduced (using the splining algorithm splinevect [Cantarella, J. *vecttools*. (2012) Available at http://www.jasoncantarella.com/wordpress/software/vecttools/]) so that their number of vertices was approximately equal to the minimum ropelength. We then used Ridgerunner to tighten the configurations. The KnotPlot configurations are freely available from the software KnotPlot [Scharein, R. G. *KnotPlot*. (1998) Available at: http://www.knotplot.com]. The random configurations were created using a variation of the Hedgehog algorithm^39^. We generated 100,000 configurations with 100 edges and the knot types were determined using the techniques described in the previous paragraph.

## Author Contributions

K.C.M., E.J.R. and A.S. conceived the project. E.J.R. performed the numerical analysis. K.C.M., E.J.R. and A.S. discussed the results and wrote the manuscript.

## Figures and Tables

**Table 1:** Agreement between the sets of predecessor knots and the sets of subknots observed in KnotPlot and ideal configurations with increasing numbers of crossings. For most of the analyzed knots, all observed subknots in the disk matrices of KnotPlot and ideal configurations belong to the set of predecessor knots of the corresponding global knot type. However, as the crossing number increases some of the KnotPlot and ideal configurations have subknots that are not predecessor knots of the global knot type. When one considers majority subknots (i.e. subknots that achieve at least 50% frequency in some subarc using our closure algorithm), then all of these subknots belong to the sets of predecessor knots of the corresponding global knots. If one concentrates on the knot types forming predecessor knots of the first generation then they are visible as subknots in the disk matrices of the KnotPlot and ideal configurations of the corresponding global knots

	number of crossings	3	4	5	6	7	8	9	10
	# alternating knot types with predecessors	1	1	2	3	7	18	35	92
KnotPlot	all subknots ⊆ predecessors	1	1	2	3	7	16	27	58
	some subknots ∉ predecessors	0	0	0	0	0	2	8	34
	all majority subknots ⊆ predecessors	1	1	2	3	7	18	35	92
	first generation predecessors ⊆ subknot set	1	1	2	3	7	18	35	92
	all first generation predecessors ⊆ majority subknot set	1	1	2	3	7	18	32	78
	some first generation predecessors ∉ majority subknot set	0	0	0	0	0	0	3	14
Ideal	all subknots ⊆ predecessors	1	1	2	3	7	18	35	89
	some subknots ∉ predecessors	0	0	0	0	0	0	0	3
	all majority subknots ⊆ predecessors	1	1	2	3	7	18	35	92
	all first generation predecessors ⊆ subknot set	1	1	2	3	7	18	35	92
	all first generation predecessors ⊆ majority subknot set	1	1	2	3	7	18	35	92
